# Association of Nonalcoholic Fatty Liver Disease and Coronary Artery Disease with FADS2 rs3834458 Gene Polymorphism in the Chinese Han Population

**DOI:** 10.1155/2019/6069870

**Published:** 2019-11-03

**Authors:** Yingyu Xu, Zhenzhen Zhao, Shousheng Liu, Yingpu Xiao, Mengyuan Miao, Quanjiang Dong, Yongning Xin

**Affiliations:** ^1^The Affiliated Qingdao Municipal Hospital of Qingdao University, Qingdao 266000, China; ^2^Qingdao Municipal Hospital Central Laboratory, Qingdao 266000, China; ^3^Qingdao Key Laboratory of Digestive Diseases, Qingdao 266000, China; ^4^Dalian Medical University, Dalian 116000, China; ^5^Nanjing Medical University, Nanjing 210000, China; ^6^Department of Infectious Disease, Qingdao Municipal Hospital, Qingdao 266000, China

## Abstract

**Background:**

Nonalcoholic fatty liver disease (NAFLD) patients are often prone to coronary artery disease (CAD), and CAD is found to be the main cause of death in NAFLD patients. The purpose of this study was to investigate the association between fatty acid desaturase 2 (FADS2) rs3834458 polymorphism and serum FADS2 level with NAFLD and CAD in Chinese Han population.

**Materials and Methods:**

The serum level of FADS2 was detected by enzyme-linked immunosorbent assay (ELISA) in healthy people, NAFLD patients, and NAFLD patients combined with CAD (NAFLD+CAD). Polymerase chain reaction (PCR) was used to detect the genotypes of FADS2 rs3834458 in the three groups.

**Results:**

Body mass index (BMI), glucose (GLU), total cholesterol (TC), and low-density lipoprotein cholesterol (LDL-C) of the NAFLD group and the NAFLD+CAD group were higher than those of the healthy control group (*P* < 0.05); the HDL-C of the NAFLD+CAD group was significantly lower than that of the healthy people and the NAFLD group (*P* < 0.05). The serum FADS2 concentration in the NAFLD+CAD group was significantly higher than that in the NAFLD group (*P* < 0.05) and the healthy people (*P* < 0.05). There was no significant difference in genotype distribution (*χ*^2^ = 5.347, *P* < 0.497) and allele frequency (*χ*^2^ = 3.322, *P* = 0.345) between the three groups. Logistic regression analysis showed that the T allele was not an independent risk factor for CAD with NAFLD (OR = 1.62, 95% CI: 0.422-6.180).

**Conclusions:**

Serum FADS2 concentration was positively correlated with the susceptibility of NAFLD with CAD, while the polymorphism of rs3834458 was not associated with NAFLD with CAD.

## 1. Introduction

NAFLD refers to the deposition of fat in hepatocytes except for excessive alcohol consumption and other factors that affect the liver's ability to clear fat. The progressive stage of NAFLD includes nonalcoholic fatty liver (NAFL), nonalcoholic steatohepatitis (NASH), hepatic fibrosis, cirrhosis, and even hepatocellular carcinoma [1]. NAFLD has become the most common chronic liver disease, accounting for 1/4 of the global population [2]. Over the past three decades, changing lifestyles and eating habits had laid the foundation for NAFLD epidemic in Asia [3]. In China, the prevalence of NAFLD in 2000-2006 was 18.2%, 2007-2009 was 20.0%, and 2010-2013 was 20.9% [4]. NAFLD is considered to be a hepatic manifestation of metabolic syndrome (MetS) [1]. MetS is a pathological condition in which various metabolic components are abnormally aggregated, including dyslipidemia, insulin resistance, obesity, and hypertension. MetS increase the risk of developing and dying of CAD [5]. Many studies show that NAFLD can increase the risk of future coronary artery disease events' occurrence and death and NAFLD can also predict the risk for the mortality of CAD [6, 7]. In addition, some studies also find that CAD is the most important cause of death in NAFLD patients [8, 9].

Polyunsaturated fatty acids (PUFAs) have the function of anti-inflammatory, regulating blood lipids and reducing hepatic steatosis [10, 11]. The ratio of (n-6) to (n-3) in the diet has increased significantly in recent decades. At the same time, the prevalence of chronic inflammatory disease also significantly increases, such as NAFLD, CAD, and obesity [12]. Delta-5 (D5D, FADS1) and delta-6 desaturases (D6D, FADS2) are key enzymes in the metabolism of n-3 and n-6 PUFAs, which enable alpha-linolenic acid (ALA) and linoleic acid (LA) to form long-chain polyunsaturated fatty acids (LC-PUFAs) [13]. D5D and D6D are, respectively, encoded by the fatty acid desaturase 1 (FADS1) gene and the FADS2 gene. The activity of FADS2 of NAFLD patients is higher than that of the normal people, while the activity of FADS1 of NAFLD patients is lower than that of the normal people [14]. Steffen et al. found that high activity of FADS2 is associated with most cardiovascular risk factors in adolescents [15]. Compared with simple steatosis, n-3 and n-6 LC-PUFAs in the liver of NASH patients are lower, which may be related to the overexpression of FADS1 and FADS2 genes [16]. And then, Walle et al. found that NASH was associated with higher mRNA expression levels of FADS1, FADS2, and stearoyl-CoA desaturase (SCD) genes in the liver [17].

The etiology and pathogenesis of patients with NAFLD complicated with CAD have become a hot topic in medical research. In NAFLD patients, the risk factors leading to atherosclerosis and accelerating thrombosis are mainly those related to MetS, such as lipid disorder, oxidative stress, hyperinsulinemia, and insulin resistance [18]. However, the reasons are not enough to explain why not all patients with NAFLD are complicated by CAD nor can they explain the difference in the degree of vascular disease in patients with NAFLD under the same conditions of noxious stimulation and similar action time. The pathogenesis of various complications of NAFLD has not been completely elucidated, but in recent years, studies on the genetic etiology of NAFLD show that NAFLD is a multigene disease, and its complications can also be affected by the genetic background of the patients.

The genetic variation of single nucleotide polymorphism (SNP) in the fatty acid desaturase (FADS) gene cluster can affect the PUFA level from different aspects, such as changing the use of promoters and transcriptional stability, so as to change the sequence and function of proteins from the FADS gene cluster. The polymorphism of the FADS2 gene causes elevated levels of triglycerides, which can lead to CAD and acute coronary syndrome (ACS) [19, 20]. Rs3834458 indel (T/del, minor allele: deletion) is very close to the translational site of putative FADS2 gene and putative regulatory regions, such as the binding site of transcription factors, sterol regulatory binding element-1c (SREB-1c), and peroxisome proliferator-activated receptor *α* (PPAR*α*) [21, 22]. The polymorphisms and expression of FADS2 gene have never been studied in NAFLD patients complicated with CAD in Chinese Han population. This study was aimed at exploring the correlation of the FADS2 concentration in serum and FADS2 rs3834458 polymorphism in NAFLD and NAFLD with CAD patients, in order to initially screen the susceptibility genes of NAFLD combined with CAD.

## 2. Materials and Methods

### 2.1. Subjects

Informed consent was obtained from each patient included in the study. The research protocol complies with the ethical code of the Helsinki Declaration of 1975 and has been approved by the Ethics Committee. 337 subjects were recruited and divided into three groups. 113 NAFLD patients were screened according to the 2010 revised Chinese guidelines for the diagnosis and treatment of nonalcoholic fatty liver disease. 75 subjects in the NAFLD+CAD group not only met the NAFLD inclusion criteria but also met the following conditions: previous history of acute myocardial infarction or coronary angiography confirmed that at least one branch of the coronary artery had stenosis greater than or equal to 50%. A total of 149 healthy people were randomly selected from the healthy blood test population of the Han nationality in Qingdao. No abnormalities were found in the healthy people group after detailed medical history inquiry, physical examination, chest X-ray examination, standard 12-lead electrocardiogram, B-mode ultrasound examination, blood routine, urine routine and stool routine, and blood biochemical examination, and CAD, NAFLD, and other diseases were excluded.

### 2.2. Clinical Data Collection and Biochemical Testing

Clinical data of all subjects were recorded in the form of questionnaire, including age, gender, body mass index (BMI), and other general information. After fasting for 12 hours, all subjects were given 5 mL venous blood in a vacuum tube. Blood samples were tested using the original kit and biochemical analyzer (Beckman Coulter, USA), and the blood biochemical indicators were qualitatively and quantitatively analyzed by spectrophotometry. Blood biochemical indicators include blood glucose (GLU), total cholesterol (TC), triglyceride (TG), low-density lipoprotein (LDL-C), high-density lipoprotein (HDL-C), total bilirubin (TIBL), alanine aminotransferase (ALT), aspartate aminotransferase (AST), *γ*-glutamyl transferase (GGT), and alkaline phosphatase (ALP).

### 2.3. Enzyme-Linked Immunosorbent Assay (ELISA) for FADS2

FADS2 concentration in the blood sample was detected under the instruction of human FADS2 ELISA kit (EK-Bioscience, China). Successively add samples, standard samples, and HRP-labeled detection antibodies in the micropores precoated with FADS2 antibody, then incubate the micropores and thoroughly wash them. Color was developed with substrate 3,3′,5,5′-tetramethylbenzidine (TMB), which was converted to blue under the catalysis of peroxidase and finally to yellow under the action of acid. The color intensity was positively correlated with the concentration of FADS2 in the sample. The absorbance (OD value) was measured with enzyme-labeled instrument at 450 nm (Enspire, China), and the concentration of FADS2 in the sample was calculated.

### 2.4. Genotyping

Whole blood genomic DNA rapid extraction kit (BioTeKe, China) was used to extract DNA from blood samples. DNA fragments were amplified by PCR. Primer design was performed using Sequenom MassARRAY Assay Design 3.1 (Sequenom, USA), and its sequences (synthesized by Beijing Boao Biotechnology Co., Ltd.) were 5′-ACGTTGGATGACCAAGAAAGCAGAGCAGAG-3′-forward primer and 5′-ACGTTGGATGCCTTGGATTAGAGGGCTTTG-3′-reverse primer. PCR master mix 4 *μ*L (PCR master mix component: H_2_O, HPLC grade 1.850 *μ*L, PCR Buffer with 15 mM MgCl_2_ 0.625 *μ*L, MgCl_2_ 0.325 *μ*L, dNTP Mix 0.100 *μ*L, Primer Mix 1.000 *μ*L, HotStar Taq, 0.100 *μ*L) was added to 1 *μ*L of DNA and mixed thoroughly. The reaction was carried out on a PCR machine (ABI, USA), predenatured at 94°C for 4 min, denatured at 94°C for 20 s, refracted at 56°C for 30 s, extended at 72°C for 1 min, and cycled for a total of 45 times. The PCR product was subjected to single base phosphatase treatment, followed by a single base extension reaction, and finally performed resin purification. Matrix-assisted laser desorption ionization time-of-flight mass spectrometry (MALDI-TOF MS) was used to detect the difference in molecular weight between the extension product and the unstretched primer and finally determined the base at that point.

### 2.5. Statistical Analysis

The measurement data was expressed as mean ± SD, and the counting data was expressed as frequency. The Hardy-Weinberg equilibrium law was used for genetic equilibrium test. The chi-square test and the Bonferroni method were used for comparison between groups. Comparison of measurement data of normal distribution between groups was tested by homogeneity of variance. One-way ANOVA was used for comparison of multiple mean values, and the Bonferroni test was used for comparison between groups. The measurement data of skewness distribution was transformed into normal distribution data for processing. If there was no appropriate transformation method, the nonparametric test was adopted. The Kruskal-Wallis *H* test was adopted for comparison between multiple groups. To balance the confounding factors, we took a multifactorial nonconditional logistic regression analysis and calculated the superiority ratio (odds ratio, OR) and 95% confidence interval (95% confidence interval, 95% CI). *P* < 0.05 was considered statistically significant.

## 3. Results

### 3.1. Comparison of Clinical Characteristics among the Healthy Control Group, the NAFLD Group, and the NAFLD+CAD group

The general clinical data of the three groups is summarized in Table 1. There was no significant difference in gender, age, TBIL, and AST between the three groups (all *P* > 0.05). Compared with the healthy control group, the BMI, GLU, TC, LDL-C, ALT, and GGT of the NAFLD group were higher than those of the healthy control group (all *P* < 0.05); the BMI, GLU, TG, TC, LDL-C, ALT, GGT, and ALP of the NAFLD+CAD group were higher than those of the healthy control group (all *P* < 0.05). Compared with the NAFLD+CAD group, the BMI, TG, LDL-C, HDL-C, ALT, and GGT of the NAFLD group were higher than those of the NAFLD+CAD group (all *P* < 0.05). The HDL-C of the NAFLD+CAD group was lower than that of the healthy control group and the NAFLD group (*P* < 0.05).

### 3.2. Comparison of Serum FADS2 Concentrations between the Healthy Control Group, the NAFLD Group, and the NAFLD+CAD Group

**Figure 1:**
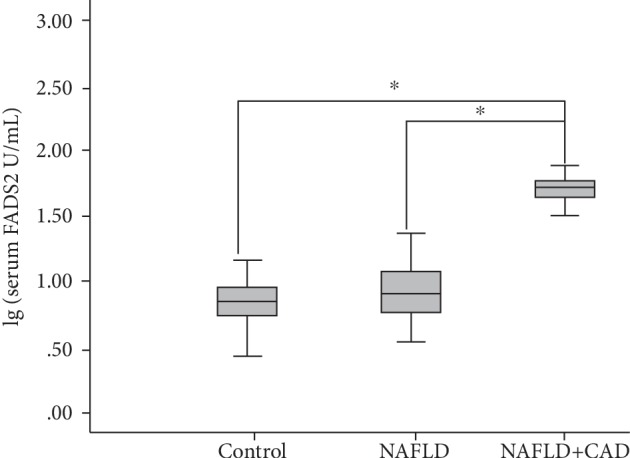
Serum FADS2 level in the healthy control group, the NAFLD group, and the NAFLD+CAD group. Control: healthy control group, NAFLD: nonalcoholic fatty liver disease group, NAFLD+CAD: Nonalcoholic fatty liver disease with coronary artery disease group, FADS2: fatty acid desaturase 2. Statistical significance was shown ^∗^*P* < 0.05.

Serum FADS2 levels were detected by ELISA. Not only was the concentration of FADS2 in serum not normal distribution but could not be converted into normal distribution; therefore, they were expressed by median (interquartile range). The difference in the concentration of FADS2 between the healthy people, the NAFLD group, and the NAFLD+CAD group was compared by the Kruskal-Wallis *H* test. The serum FADS2 level in the NAFLD+CAD group (53.72 (15.02)) is significantly higher than that in the NAFLD group (7.93 (6.09)) (*P* < 0.05) and healthy people (6.94 (3.61)) (*P* < 0.05) (Figure 1).

### 3.3. Hardy-Weinberg Genetic Balance Test of the Healthy Control Group, the NAFLD Group, and the NAFLD+CAD Group

There were no statistically significant differences between the observed frequency and the expected number of FADS2 genotypes in the healthy control group (*χ*^2^ = 1.088, *P* = 0.297), the NAFLD group (*χ*^2^ = 0.001, *P* = 0.977), and the NAFLD+CAD group (*χ*^2^ = 0.797, *P* = 0.372), indicating that the observed frequency and expected number of FADS2 genotypes in each group were consistent. This suggested that the population in this study conformed to the Hardy-Weinberg law of genetic balance. The data was reliable and representative.

### 3.4. Comparison of Genotype Distribution and Allele Frequency between the Healthy Control Group, the NAFLD Group, and the NAFLD+CAD Group

Genotype identification was performed on all subjects, and there were three FADS2 rs3834458 genotypes, including T/T, T/del, and del/del (del denotes the deletion of T allele). There was no statistically significant difference in genotype distribution among the three groups (*χ*^2^ = 2.459, *P* = 0.652) nor was there statistically significant difference in allele frequency among the three groups (*χ*^2^ = 0.688, *P* = 0.709) (Table 2). The genotype distribution and allele frequency of FADS2 rs3834458 had no significant correlation with NAFLD and NAFLD with CAD susceptibility.

### 3.5. The Results of the Independent Risk Factor of the CAD with NAFLD using the Logistic Regression Analysis

In order to correct the effects of confounding factors, all NAFLD patients were classified with or without CAD. Sex, age, BMI, GLU, TG, TC, LDL-C, HDL-C, and allele were used as independent variables. In Table 3, BMI (*P* = 0.003), GLU (*P* = 0.006), and TC (*P* = 0.022) got statistical significance, while T allele got no statistical significance (OR = 1.62, 95% CI: 0.422-6.180, *P* = 0.483), suggesting that T allele is not an independent risk factor for NAFLD combined with CAD.

## 4. Discussion

FADS2 and FADS1 are membrane-bound enzymes and are also rate-limiting enzymes that catalyze the synthesis of LC-PUFAs [23, 24]. FADS2 catalyzes ALA (18:3n-3) and LA (18:2n-6) convert to stearidonic acid (STD, 18:4n-3) and *γ*-linolenic acid (GLA, 18:3n-6). The PUFAs mentioned above play an important role in maintaining membrane integrity and regulating cell signal conduction, which affect the development of various diseases [25]. CAD [26], MetS [27], and other diseases have been shown to be related to the irregularity of the fatty acid composition of the membrane. NAFLD is considered as the liver manifestation of MetS. Therefore, NAFLD may also be related to the fatty acid composition of the membrane. Moreover, some studies prove that CAD and ACS are closely related to MetS [28, 29]. Through the detection of serum FADS2 levels in different populations, our study found that FADS2 was significantly higher in NAFLD patients with CAD than those with NAFLD alone and healthy people, further proving the important role of FADS2 in NAFLD complicated CAD. FADS2 may be a factor contributing to the progression of CAD. High expression of FADS2 may promote the occurrence and development of CAD in NAFLD patients.

This study found the importance of FADS2 in patients with NAFLD and CAD. Therefore, further study on the effect of gene variation on the regulation of FADS2 content or activity can help us better understand how these factors mediate the susceptibility to NAFLD and CAD. There were studies that show that the enzyme encoded by FADS2 gene can convert LA and ALA into GLA and STD, respectively [30]. FADS2 gene polymorphism is associated with MetS, and NAFLD as a component of Mets is associated with increased risk of CAD [6, 31–33]. Therefore, we speculate that the FADS2 gene polymorphism is associated with NAFLD and CAD.

In this study, univariate analysis was used to find that BMI, GLU, TC, and LDL-C in the NAFLD group and the NAFLD+CAD group were significantly higher than healthy controls; the HDL-C of the NAFLD+CAD group was significantly lower than that of the healthy control group and the NAFLD group. This indicated that high BMI, high GLU, high TC, high LDL-C, and low HDL-C were cardiovascular risk factors, which was consistent with previous research results [34, 35]. To further explore the independent risk factors for NAFLD combined with CAD, we performed multivariate logistic regression analysis. We found that BMI, GLU, and TC were independent risk factors for NAFLD combined with CAD, while the T allele of FADS2 rs3834458 was not an independent risk factor for NAFLD with CAD.

It remains unclear how FADS2 rs3834458 polymorphism affects the occurrence and development of NAFLD and CAD. As FADS2 rs3834458 may affect gene transcription and translation, it may eventually change the expression level of FADS2 in the body or local organs. Previous studies showed that FADS2 was associated with many biological processes and several diseases including NAFLD and CAD, and this association could be mediated by regulating LC-PUFA metabolism. In addition, dietary supplementation of LC-PUFAs can modulate hepatic steatosis. For example, supplementation with EPA and DHA may inhibit hepatic steatosis by inhibiting sterol regulatory element-binding proteins [36]. It was reported that T insertion decreases the activity of the promoter by about 6 times when the fibroblast cell line of NIH/3T3 mice was used [21], while human hepatocellular carcinoma cell line HepG2 was used for the study, there was no effect on T insertion of promoter activity [37]. Gregory et al. found that the enhanced activity of FADS2 related to the T allele may be the product of other genetic variants that were out of balance with the T allele or even the result of haploid effect [38]. This study did not find that FADS2 rs3834458 polymorphism is associated with NAFLD with CAD. The reason may be that the FADS2 rs3834458 with other FADS2 polymorphisms or with other genes formed unbalanced linkage inheritance. However, we only studied FADS2 rs3834458 polymorphism and ignored the interaction of the polymorphism. It is also possible that the activity of enzymes is not only dependent on the gene structure but also the gene transcriptional regulation and the substrates may have certain effects on the activity and expression of enzymes. Of course, the small sample size may also affect the results of this study.

## 5. Conclusions

This study did not find that the FADS2 rs3834458 polymorphism was associated with NAFLD and CAD. However, the expression product of FADS2 gene was significantly increased in patients with NAFLD combined with CAD. This indicated that the FADS2 was associated with NAFLD and CAD, while the polymorphism of FADS2 rs3834458 was not related to NAFLD with CAD. If the susceptibility genotype of NAFLD with CAD can be found, it will be helpful for the early diagnosis and prevention of cardiovascular complications in NAFLD patients. Meanwhile, it will be helpful to further reveal the molecular genetic mechanism of NAFLD with CAD.

## Figures and Tables

**Table 1:** Comparison of clinical characteristics among the control group, the NAFLD group, and the NAFLD+CAD group.

Indicators	Control	NAFLD	CAD+NAFLD	*P*
Cases (male/female)	149 (75/74)	113 (59/54)	75 (38/37)	0.927
Ages (years)	50.80 ± 12.15	50.93 ± 2.75	52.27 ± 4.06	0.442
BMI (kg/m^2^)	23.75 ± 3.56	26.44 ± 2.53	25.17 ± 2.56	<0.001
GLU (mmol/L)	4.56 (1.11)	4.84 (0.67)	5.32 (1.43)	<0.001
TBIL (*μ*mol/L)	13.17 ± 5.36	13.02 ± 4.65	14.33 ± 7.20	0.242
ALT (U/L)	17.66 (11.16)	23.62 (21.20)	22.63 (18.03)	<0.001
AST (U/L)	20.71 (6.44)	21.64 (7.44)	20.96 (18.43)	0.141
GGT (U/L)	19.59 (14.54)	32.55 (27.24)	25.15 (16.65)	<0.001
ALP (U/L)	73.99 ± 23.58	70.26 ± 17.35	85.67 ± 21.83	<0.001
TG (mmol/L)	1.11 (0.75)	1.67 (1.17)	1.46 (1.21)	0.031
TC (mmol/L)	3.47 ± 0.80	5.51 ± 0.84	4.47 ± 1.19	<0.001
LDL-C (mmol/L)	2.06 ± 0.56	3.27 ± 0.59	2.68 ± 0.92	<0.001
HDL-C (mmol/L)	1.33 ± 0.48	1.23 ± 0.21	1.05 ± 0.28	<0.001

Control: healthy control; NAFLD: nonalcoholic fatty liver disease; NAFLD+CAD: NAFLD patients with coronary artery disease. The data of normal distribution were expressed as mean ± SD; the data of nonnormal distribution were expressed as median (interquartile range). BMI: body mass index; GLU: glucose; TBIL: total bilirubin; ALT: alanine aminotransferase; AST: aspartate aminotransferase; GGT: *γ*-glutamyl transferase; ALP: alkaline phosphatase; TG: triglyceride; TC: total cholesterol; LDL-C: low-density lipoprotein cholesterol; HDL-C: high-density lipoprotein cholesterol.

**Table 2:** Comparison of genotype distribution and allele frequency among the control group, the NAFLD group, and the NAFLD+CAD group.

Groups		Control *n* = 149	NAFLD *n* = 113	CAD+NAFLD *n* = 75	*χ* ^2^	*P*
Genotypes (%)	T/T	75 (50.03)	58 (51.32)	44 (58.67)	2.459	0.652
	T/del	65 (43.62)	46 (40.71)	25 (33.33)		
	del/del	9 (6.04)	9 (7.96)	6 (8.00)		
Alleles (%)	T	215 (72.15)	162 (71.68)	113 (75.33)	0.688	0.709
	del	83 (27.85)	64 (28.32)	37 (24.67)		

Control: healthy control; NAFLD: nonalcoholic fatty liver disease; NAFLD+CAD: NAFLD patients with coronary artery disease.

**Table 3:** The results of the independent risk factor analysis of the CAD with NAFLD.

Variance	OR	95% CI	*P*
Male	1.23	0.576-2.624	0.594
Age	1.12	0.996-1.261	0.057
BMI	0.79	0.673-0.924	0.003
GLU	1.41	1.102-1.799	0.006
TG	0.94	0.686-1.289	0.703
TC	0.17	0.036-0.774	0.022
LDL-C	4.04	0.585-27.986	0.157
HDL-C	0.17	0.020-1.464	0.107
T allele	1.62	0.422-6.180	0.483

BMI: body mass index; GLU: glucose; TG: triglyceride; TC: total cholesterol; LDL-C: low-density lipoprotein cholesterol; HDL-C: high-density lipoprotein cholesterol; OR: odds ratio; 95% CI: 95% confidence interval.

## Data Availability

The data used to support the findings of this study are available from the corresponding author upon request.
